# Meiotic gene activation in somatic and germ cell tumours

**DOI:** 10.1111/andr.12628

**Published:** 2019-05-17

**Authors:** J. Feichtinger, R. J. McFarlane

**Affiliations:** ^1^ Division of Cell Biology, Histology and Embryology Gottfried Schatz Research Center for Cell Signaling, Metabolism and Aging Medical University of Graz Graz Austria; ^2^ OMICS Center Graz BioTechMed Graz Graz Austria; ^3^ North West Cancer Research Institute School of Medical Sciences Bangor University Bangor Gwynedd UK

**Keywords:** genome stability, germ cell tumour, meiosis, oncogenesis

## Abstract

**Background:**

Germ cell tumours are uniquely associated with the gametogenic tissues of males and females. A feature of these cancers is that they can express genes that are normally tightly restricted to meiotic cells. This aberrant gene expression has been used as an indicator that these cancer cells are attempting a programmed germ line event, meiotic entry. However, work in non‐germ cell cancers has also indicated that meiotic genes can become aberrantly activated in a wide range of cancer types and indeed provide functions that serve as oncogenic drivers. Here, we review the activation of meiotic factors in cancers and explore commonalities between meiotic gene activation in germ cell and non‐germ cell cancers.

**Objectives:**

The objectives of this review are to highlight key questions relating to meiotic gene activation in germ cell tumours and to offer possible interpretations as to the biological relevance in this unique cancer type.

**Materials and Methods:**

PubMed and the GEPIA database were searched for papers in English and for cancer gene expression data, respectively.

**Results:**

We provide a brief overview of meiotic progression, with a focus on the unique mechanisms of reductional chromosome segregation in meiosis I. We then offer detailed insight into the role of meiotic chromosome regulators in non‐germ cell cancers and extend this to provide an overview of how this might relate to germ cell tumours.

**Conclusions:**

We propose that meiotic gene activation in germ cell tumours might not indicate an unscheduled attempt to enter a full meiotic programme. Rather, it might simply reflect either aberrant activation of a subset of meiotic genes, with little or no biological relevance, or aberrant activation of a subset of meiotic genes as positive tumour evolutionary/oncogenic drivers. These postulates provide the provocation for further studies in this emerging field.

## Introduction

Human gametogenesis generates spermatozoa and oocytes that may ultimately fuse to form a zygote. Whilst the human male and female gametogenic programmes differ considerably in their timing and context, with a defined number of oocytes generated only in the foetal ovaries and spermatozoa produced continually in the testis of sexually competent, post‐pubescent males, both share the requirement to undergo a reduction chromosomal segregation from the diploid state during meiosis (Spiller *et al*., 2012; Feng *et al*., 2014; Griswold, 2016; Nagaoka & Saitou, 2017; Sanders & Jones, 2018). The extent to which male and female meiosis differs in humans has undergone relatively little investigation, mostly due to technical/ethical issues and limitations of reconstitution of mammalian gametogenic programmes *in vitro*. Given this, much of what is known about the regulation of meiotic initiation and the mechanisms of meiotic chromosomes segregation comes from studies in model eukaryotes, particularly murine systems, with a greater emphasis on spermatogenesis (Griswold, 2016). Thus, much of our understanding of these processes is extrapolative in nature. Despite these constraints, it is becoming clear that activation of meiotic genes (and other germ line genes) occurs during oncogenesis and that these genes provide functions for both initiation and maintenance of the cancerous state in a range of cancer types (Simpson *et al*., 2005; Fratta *et al*., 2011; Rousseaux *et al*., 2013; Lafta *et al*., 2014; McFarlane *et al*., 2014, 2015; Whitehurst, 2014; Nielsen & Gjerstorff, 2016). It is an emerging possibility that meiotic deregulation is a feature of germ cell (GC) tumours (for example, see Jørgensen *et al*., 2013). The extent of the contribution to distinct aspects of oncogenesis by meiotic genes, whose expression is normally restricted to gametogenesis, has only undergone limited scrutiny to date. Moreover, their role in GC tumour development and progression is complicated by the fact that GC tumours are associated with the gametogenic tissues. So, distinguishing aberrant activation of one or more meiotic and germ line genes from attempted inappropriate entry into a full meiotic programme possesses important, yet challenging questions; for example:

- Does activation of meiotic genes in GC tumours indicate that these cells are attempting a true germ line meiotic entry?
- Can analysis of expression of a subset of selected meiotic genes be used as markers to indicate a *bona fide* meiotic entry signalling network?
- Or, are these genes being activated independently of a full meiotic entry programme? And if so, what regulates their activation?
- Do these genes provide meiotic‐like functions that contribute to oncogenic maintenance, progression and therapeutic resistance in GC tumours, as they do in other cancer types?

Here, we provide insight from recent studies on the role of meiotic genes in a wide range of cancers. Whilst limited data negate addressing the emerging questions associated with GC cancers, we aim to offer the context in which these questions should be embedded.

## Meiosis: A Brief Overview

**Figure 1:**
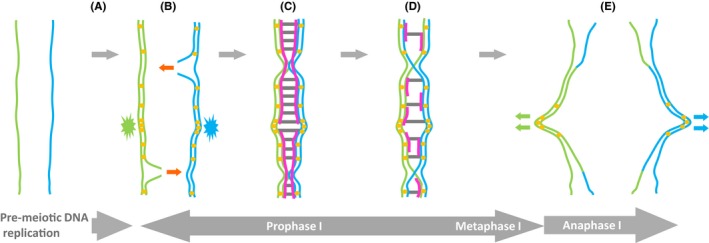
Schematic of chromosome dynamics during the reductional segregation of meiosis I. The progression from left to right shows a pair of homologous chromosomes (green and blue) undergoing pre‐meiotic DNA replication (A), through to anaphase I (E). (A) During pre‐meiotic DNA replication, cohesion is established between sister chromatids (yellow dots). This is mediated by a ring‐shaped complex termed cohesin. In meiosis, some chromosomal cohesin complexes contain meiosis‐specific subunits, some of which can be activated during oncogenesis. Cohesin is enriched at the centromeric regions (denoted by the starburst shapes). (B) Early in prophase I, homologous chromosomes align with one another and meiotic recombination is initiated by the generation programmed of DNA double‐strand breaks (DSBs). DSBs occur predominantly at specific genomic loci termed hot spots (illustrated by the red arrow). Meiosis‐specific mechanisms direct homologous recombination to repair the DSBs preferentially via an inter‐homologue route, as opposed to an inter‐sister chromatid route (red arrows). (C) This inter‐homologue recombination results in the formation of stable homologous recombination intermediates (illustrated by the constriction points) and the formation of a bivalent. A continuous proteinaceous ladder‐like structure forms between the synapsed homologues called the synaptonemal complex (SC). The SC comprises axial structures associated with the cohesin complex (magenta lines) on each homologue and these are conjoined by a central element making the rungs of the ‘ladder’ (horizontal grey lines). The SC comprises many meiosis‐specific factors, some of which can become activated during oncogenesis, such as SYCP3, a component of the axial structures of the SC. (D) Late in prophase I, the SC starts to break down and homologous recombination intermediates (Holliday junctions) dissociate to give an obligate crossover in each arm of the bivalent. (D–E) Cells transition through metaphase I during which time the spindle forms monopolar kinetochore associations with the centromeres to give a reductional segregation. Monopolarity is dependent on meiosis‐specific cohesin subunit REC8. The metaphase I‐to‐anaphase I transition requires cleavage of sister chromatid cohesion in the arms of the bivalent, which releases crossed over chromosome arms. Centromeric cohesion is maintained in metaphase I enabling the monopolar migration of the sister centromeres (E). Sister centromere cohesion is maintained to orchestrate inter‐sister associations at metaphase II, where sister centromeres now form bipolar spindle associations permitting an equational segregation during anaphase II (not shown).

After arrival of primordial germ cells (PGCs) at the developing gonad, the cells undergo extensive epigenetic reprogramming, and development is directed either towards ovaries or testis depending on the presence or absence of a functioning *SRY* gene, which is normally located on the Y chromosome (Witchel, 2018). There are pronounced differences in regulation and timing of gametogenesis in females and males, but both require a meiotic chromosome segregation programme to drive haploidization; in the foetal ovaries, a defined number of oocytes enter prophase I of meiosis I, whereas in the foetal testes, meiotic entry is inhibited until puberty and spermatozoa are subsequently produced continually (Jørgensen & Rajpert‐De Meyts, 2014). However, during the general process of meiosis diploid germ line progenitor cells undergo a single round of pre‐meiotic DNA replication followed by two chromosome segregation events, meiosis I (reductional) and meiosis II (equational), ultimately creating haploid gametes (Zickler & Kleckner, 1999) (Fig. 1 shows a schematic of the meiosis I reductional segregation).

The hormonal cues and signalling cascades for human meiotic entry are poorly delineated, but it is now thought that meiotic entry is initiated by retinoic acid (RA) coupled with *STRA8* (Stimulated by Retinoic Acid 8) gene expression (Feng *et al*., 2014; Ma *et al*., 2018). In foetal ovaries, RA and STRA8 induce the meiotic initiation programme, whereas the entry into meiosis in males is thought to be inhibited by the expression of the *CYP26B* gene, which encodes a RA‐degrading enzyme. Moreover, a number of important regulatory players have been identified in recent years, including DAZL, FGF9, NANOS2/3 and DMRT1 (Jorgensen *et al*., 2012; Spiller *et al*., 2012; Zhang & Zarkower, 2017).

**Figure 2:**
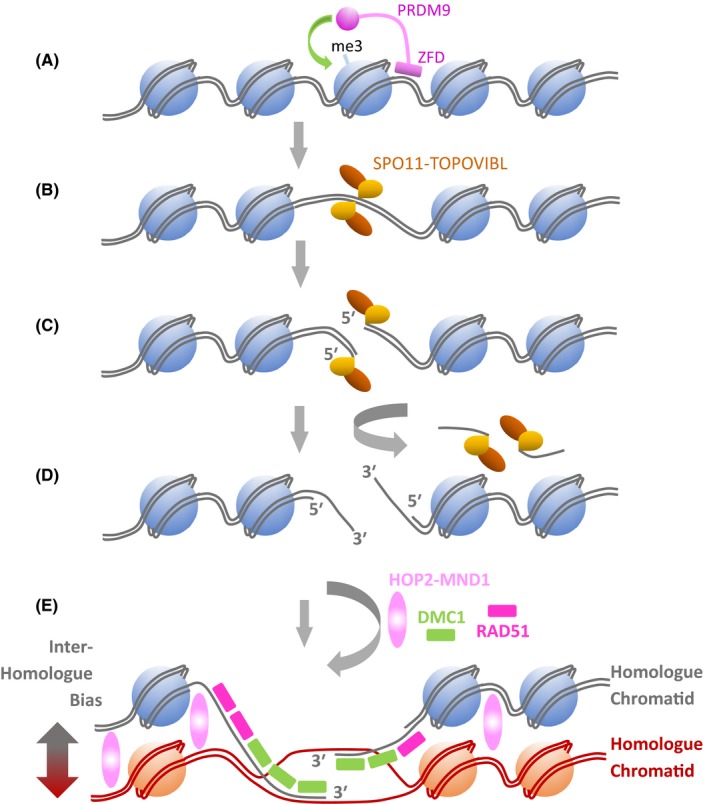
Schematic of the initiation of meiotic inter‐homologue recombination at hot spot loci. (A) Chromatin is specifically marked by the hot spot activator PRDM9. PRDM9 is a histone methyltransferase that recognizes specific DNA sequence motifs via a zinc finger domain (ZFD). Association with hot spot motifs is followed by local methylation of histones to ‘open’ the chromatin making the DNA accessible to double‐strand break (DSB) mediators. (B) An atypical topoisomerase‐like complex (SPO11‐TOPIVIBL) associates with the DNA, and the SPO11 moiety introduces a programmed DSB. (C–D) SPO11 remains covalently bound to each 5′ end of the DSB site. The 5′ end covalently bound to SPO11 is processed away exposing single‐stranded (ss) DNA with a free 3′ end. (D–E) The 3′ ssDNA ends associate with the recombinases RAD51 and DMC1, the latter being meiosis‐specific. Via interaction with a number of factors, such as HOP2‐MND1, the recombinases (RAD51/DMC1) mediate inter‐chromatid recombination with a meiosis‐specific inter‐homologue bias. Subsequent processing results in the stabilization of these early inter‐homologue intermediates, and a bivalent is formed (see B–D in Fig. 1).

During pre‐meiotic DNA replication, newly formed sister chromatids remain connected via the actions of a ring structure termed cohesin that encircles both sister chromatids (Ishiguro, 2018) (Fig. 1A,B). Following this, the homologous chromosomes undergo a dramatic juxtapositioning to initiate pairing and alignment. During this period, a specific meiotic recombination programme is initiated by the generation of DNA double‐stranded breaks (DSBs) in one chromatid of one of the homologues (Humphryes & Hochwagen, 2014; Hunter, 2015; Gray & Cohen, 2016) (Fig. 1B). This is initiated by a topoisomerase VI‐like complex, which consists of SPO11 and TOPOVIBL (C11orf80) (Robert *et al*., 2016) (Fig. 2B,C). The SPO11 subunit remains covalently bound to the 5′ end of each side of the break, both of which are subsequently resected to generate a stretch of single‐stranded DNA with a free 3′ end (Fig. 2C,D). This provides the substrates for the recombinase RAD51 and its meiosis‐specific paralogue DMC1, enabling them to mediate the strand invasion of a homologous duplex (Hunter, 2015) (Fig. 2E).

Meiotic recombination is not initiated uniformly throughout the genome, with some loci being more prone to serve as recombination initiation sites (Fig. 1B); these are referred to as meiotic recombination hot spots, and there are regions where the chromatin takes on a more open, permissive configuration enabling the SPO11‐TOPOVIBL complex to access the DNA (Wahls & Davidson, 2010; Tock & Henderson, 2018). Hot spot chromatin activation is driven by meiosis‐specific factors such as the zinc finger histone methyltransferase, PRDM9 (Grey *et al*., 2018; Paigen & Petkov, 2018) (Fig. 2A).

Importantly, unlike mitosis, where DSB repair mediated by homologous recombination is mostly channelled down an inter‐sister chromatid route, the repair of programmed meiotic DSBs occurs with a bias to inter‐homologue strand invasion mediated by an additional group of meiosis‐specific factors, which include HOP2 (PSMC3IP)‐MND1 (Hunter, 2015) (Figs 1B,C and 2E). Some recombination intermediates generated via this inter‐homologue route dissociate and do not progress to make chromosome crossovers; others, however, ultimately form Holliday junctions and can be processed to generate chiasmata structures in the bivalent and the chromosomal crossovers that serve as a major evolutionary driver (Hunter, 2015) (Fig. 1D).

As the recombination‐dependent bivalents align on the metaphase I plate, the centromeres of sister chromatids form monopolar spindle associations that are unique to meiosis I and are required to drive the reductional segregation at this stage (Zickler & Kleckner, 1999) (Fig. 1D,E). Loss of sister cohesion in the arm regions of chromosomes, but not the centromeric regions, occurs on entry into anaphase I permitting reductional segregation, as this disrupts the bivalent, yet permits inter‐sister cohesion to be maintained at the centromeres (Fig. 1E). During meiosis II, centromeric cohesion is broken down and an equational segregation ensues (Ishiguro, 2018).

## Activation of Meiotic Chromosome Regulators in Non‐Germ Cell Cancers

Germ line gene activation, including meiotic genes, appears to be a common feature in a range of human neoplastic conditions (McFarlane *et al*., 2014, 2015; McFarlane & Wakeman, 2017). If applied to mitotically proliferating cells, the specific molecular mechanisms driving reductional segregation in gametogenic meiosis I could be considered to be deleterious and potentially highly oncogenic. Examples include the genesis of DSBs, the modulation of inter‐homologue repair of DSBs that could drive loss of heterozygosity, the alteration of the transcriptional landscape, the epigenetic activation of recombination hot spot loci and the monopolar orientation of sister chromatid centromeres. Evidence is starting to emerge that meiosis‐like activities mediated by unscheduled activation of meiotic genes do indeed contribute to oncogenesis, but there are some unexpected ways in which these activities make their contribution, challenging intuitive hypotheses for roles of meiotic gene activity in cancer cells (McFarlane & Wakeman, 2017). This is particularly apparent when applied to GC tumours where activation of meiosis‐specific genes might be taken to imply meiotically primed cells are attempting a programmed entry into meiosis, albeit a flawed one (Jørgensen *et al*., 2013). This might indeed be the case, but the evidence from studies of oncogenic roles in other cancers and model systems should engender consideration of other possibilities for the causes and roles of meiotic gene activation in GC tumours.

### The meiotic recombination initiators in cancer

Cancer/testis (CT) genes are a class of gene that has expression normally restricted to the testis of adult males, but which become active in some, if not even all cancers (Simpson *et al*., 2005; Whitehurst, 2014; Gibbs & Whitehurst, 2018). SPO11, the key mediator of meiotic DSB formation, and PRDM9, the histone methyltransferase activator of a subgroup of meiotic recombination hotspots, are both encoded for by meiosis‐specific genes that have been reported to be CT genes (Koslowski *et al*., 2002; Feichtinger *et al*., 2012). To date, no direct evidence has been put forward to indicate human SPO11 has oncogenic function(s), but remarkable insight into a potential oncogenic role comes from seminal work in *Drosophila melanogaster* (Janic *et al*., 2010; Rossi *et al*., 2017). It has been demonstrated that the formation of *D. melanogaster* larval brain tumours can be generated when lava carrying temperature‐sensitive alleles of the *l(3)mbt* gene are shifted to the non‐permissive condition (Janic *et al*., 2010). Transcriptional profiling of these tumours demonstrated that they activated a large number of germ line genes, some of which were required for tumour formation (Janic *et al*., 2010; Rossi *et al*., 2017). Interestingly, activation of a similar gene profile is also observed in a number of human cancers (Feichtinger *et al*., 2014). Two of the fly genes required for *l(3)mbt* tumour formation were *mei‐W68* and *mei‐P22*, the orthologues of human *SPO11* and *TOPOVIBL,* respectively (Rossi *et al*., 2017). This work was extended to demonstrate that the oncogenic role of the *D. melanogaster* orthologue of SPO11 (Mei‐W68) for tumour formation could be suppressed, to some degree, by ionizing irradiation, suggesting that the function that Mei‐W68 mediates is DSB‐associated (although this remains to be fully determined as irradiation does activate a small cohort of additional genes which could potentially suppress the need for a non‐DSB‐forming function of Mei‐W68) (Rossi *et al*., 2017).

The meiosis‐specific histone methyltransferase gene *PRDM9* has also been reported to be activated in cancers (Feichtinger *et al*., 2012). The murine orthologue of PRDM9, Meisetz, not only serves as a meiotic recombination activator (Fig. 2A), but also serves as a transcriptional activator for a specific subgroup of genes in meiosis (Hayashi *et al*., 2005). Overexpression of human *PRDM9* in HEK293T cells can also activate the upregulation of a number of human genes, indicating that expression of one meiosis‐specific epigenetic regulator can alter the transcriptional landscape of human cells (Altemose *et al*., 2017). This opens up a number of possibilities in terms of transcriptional deregulation in cancers by single germ line/meiotic regulatory gene.

However, new evidence has recently emerged to suggest a link between meiotic recombination hot spots and genome stability (Houle *et al*., 2018; Kaiser & Semple, 2018). PRDM9 has an affinity for specific genomic sequences mediated by the PRDM9 zinc finger domain (Grey *et al*., 2018; Paigen & Petkov, 2018) (Fig. 2A). Analysis of these binding sites in human cancers indicates that these are associated with sites of genome instability in cancers (Houle *et al*., 2018; Kaiser & Semple, 2018). Intuitively, this might suggest that they are causing a SPO11‐TOPOVIBL‐mediated break that becomes the site for chromosomal instabilities, but this would require the coordination of a number of meiosis‐specific factors (e.g. SPO11 and PRDM9) to act in concert to generate a break, which seems overly complex. An additional and possibly more plausible explanation is that PRDM9 generates chromatin regions that become more fragile for other reasons, such as causing unscheduled barriers to DNA replication which are known to be major initiators of genome instability during oncogenesis (Blumenfeld *et al*., 2017; Zhang *et al*., 2018). Exactly what the mechanism of oncogenic *PRDM9* is remains open to experimental scrutiny, but another feature to consider is the possibility that this serves to assist tumour evolution by garnering instability; it is widely accepted that tumours which have greater propensity to undergo high rates of genomic change and instability are more amenable to evolving therapeutic resistance (Sansregret *et al*., 2018). Whilst it remains to be determined whether enhanced tumour evolution is a feature of *PRDM9* activation, there is evidence that activation of other meiotic genes does indeed influence therapeutic evasion (see below).

Recently, another CT gene involved in SPO11‐mediated recombination regulation in mammals, *TEX19*, has been reported to be required for maintaining the proliferative state of cancer cells (Planells‐Palop *et al*., 2017). The functional nature of human TEX19 in cancer cells remains unknown, but the *TEX19* gene appears to be extensively activated in a range of cancer type (Feichtinger *et al*., 2012; Zhong *et al*., 2016). In the mouse, there are two *TEX19* orthologues, *Tex19.1* and *Tex19.2*, although the former paralogue is thought to be the functional orthologue of human *TEX19* (Kuntz *et al*., 2008; Ollinger *et al*., 2008). Murine Tex19.1 appears to regulate a number of germ line biological functions, most likely via a defined association with the murine Ubr2 E3 ubiquitin ligase, and these include regulation of LINE1 transposition and the initiation of SPO11 meiotic recombination (Yang *et al*., 2010; Reichmann *et al*., 2013; Tarabay *et al*., 2013; Crichton *et al*., 2017, 2018; MacLennan *et al*., 2017). *TEX19* is mammalian‐specific and so the *D. melanogaster l(3)mbt* tumour studies do not shed any light on whether *TEX19* is required early in the oncogenic programme, as well as being required to maintain the proliferative status of cancer cells, although preliminary analysis of human tumour progression arrays suggests that it is activated early in the oncogenic programme (Planells‐Palop *et al*., 2017).

### Meiosis I higher order chromosome modulators in cancers

Synapsis in meiosis I is marked by the formation of a proteinaceous, ladder‐like structure known as the synaptonemal complex (SC), which interconnects homologous chromosomes (Cahoon & Hawley, 2016) (Fig. 1C). Whilst the exact role of the SC remains unclear, it is thought to modulate crossover control, although it is not obligate, as some eukaryotes can mediate a reductional meiosis I, with crossing over, in the absence of a *bona fide* SC (Zickler & Kleckner, 1999). The formation of the SC is mediated by proteinaceous axial structures, which develop on sister chromatid pairs. Axial components include SYCP3, the presence of which is frequently used as a molecular marker for meiotic progression in GC tumours (for example, see Jørgensen & Rajpert‐De Meyts, 2014). The linear axial structures formed on each homologue come together via the development of a central cross‐linking structure containing the conserved SYCP1 proteins to form the mature SC (Cahoon & Hawley, 2016). In addition to axial structural components, such as SYCP3, other factors are required to ensure that inter‐homologue recombination and SC formation are correctly orchestrated, such as the HORMA domain protein HORMAD, which also associates with the axis (Wojtasz *et al*., 2009; Shin *et al*., 2010; Daniel *et al*., 2011).

**Figure 3:**
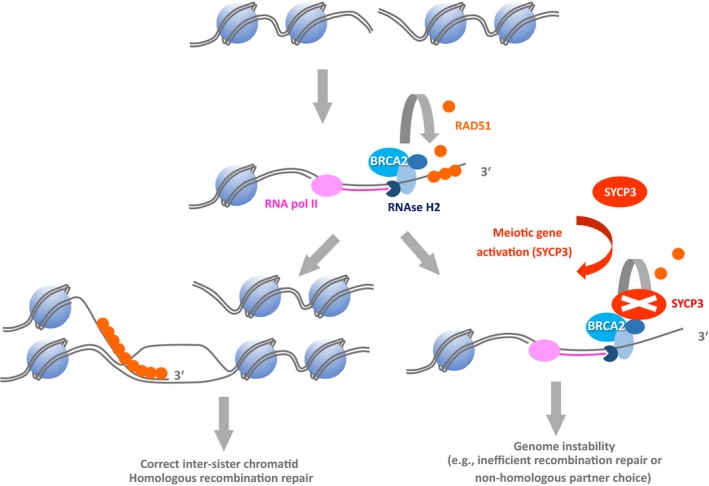
Model of an example of a meiosis‐specific recombination regulator altering genome dynamics in a non‐meiotic cell: SYCP3 disruption of BRCA2 homologous recombination function. BRCA2 is required to facilitate the loading of the RAD51 recombinase, which is required for the strand invasion step of inter‐sister chromatid DNA double‐strand break (DSB) repair in somatic, non‐meiotic cells. A normal cellular response to a DSB requires RNA polymerase II to mediate the *de novo* transcription of a damage‐inducible long non‐coding RNA (magenta strand) at the site of the DSB. This assists the resectioning of the DSB to expose single‐stranded (ss) DNA with a free 3′ end and the recruitment of a cluster of end‐processing factors, including BRCA2 and RNase H2, the latter processing the RNA:DNA hybrid generated by RNA polymerase II to permit RAD51 loading onto the ssDNA end. BRCA2 functions to assist the loading of the RAD51 recombination onto ssDNA and prevents aberrant loading onto double‐stranded DNA. Under normal circumstances, effective RAD51 loading facilitates the next stages in DSB repair. However, when the meiotic factor SYCP3 is present and active, it associates with BRCA2, inhibiting its RAD51 regulatory capability, disrupting normal DSB repair and potentially resulting in oncogenic genome instability, including aberrant repair partner choice. Adapted from Hosoya *et al*. (2011).

Recent studies have demonstrated that both SYCP3 and HORMAD1 can potentially provide oncogenic function and have the capacity to do so outside the context of a full meiotic programme. Given this, it is important to view GC tumour biology in the full light of the capabilities of these meiotic proteins (see below). SCYP3 produced outside the meiotic context has been shown to disrupt the activity of the tumour‐suppressing recombination regulator BRCA2 and can modulate the strand invasion capabilities of both the RAD51 and DMC1 (meiosis‐specific) recombinases that drive homologous recombination (Hosoya *et al*., 2011; Kobayashi *et al*., 2017) (Fig. 3). Overexpression of *SCYP3* can also drive ploidy changes (Hosoya *et al*., 2011), and, coincidently, ploidy changes are a key feature of a number of GC tumours (for example, see Jørgensen *et al*., 1997). Aberrant *SYCP3* expression has also been associated with a distinct range of cancers, and it can serve as a prognostic marker in both cervical and non‐small‐cell lung cancers (Chung *et al*., 2013; Cho *et al*., 2014a; Kitano *et al*., 2017).

Additionally, important roles for HORMAD1 in controlling oncogenic recombination pathways and therapeutic resistance have emerged (Watkins *et al*., 2015; Gao *et al*., 2018; Nichols *et al*., 2018; Wang *et al*., 2018). Two recent studies have demonstrated that *HORMAD1* expression is linked to poor prognosis and drives genotoxic (including irradiation) and oxidative stress resistance in lung adenocarcinomas (Gao *et al*., 2018; Nichols *et al*., 2018). It is postulated that this is mediated by HORMAD1 promoting efficient re‐sectioning of DSBs, making the repair of therapeutic‐induced damage more effective in cancer cells, a process that could potentially give these cells a greater evolutionary capacity. Consistent with this, HORMAD1 has also been implicated in resistance to the poly‐ADP ribose polymerase (PARP) inhibitor rucaparib in basal‐like breast cancers (Wang *et al*., 2018). PARP inhibitors block the efficient repair of nicks in double‐stranded DNA ultimately leading to the formation of more DSBs; in cancer cells with deficiencies in DSB repair, such as *BRCA2*‐negative cells, they become sensitive to rucaparib as it is synthetically lethal with unrepaired DSBs (due to BRCA2 deficiency, for example). It is proposed that HORMAD1 mediates rucaparib resistance by activating a distinct DSB repair pathway that suppresses the synthetic lethality of rucaparib and failed DSB repair. Indeed, Watkins *et al*. (2015), who first demonstrated a recombination‐associated function for HORMAD1 in cancer cells, have postulated that aberrant expression of *HORMAD1* disrupts the normal homologous recombination repair pathway driven by the recombinase RAD51 resulting in a preference for a distinct DSB repair pathway, one that makes triple‐negative breast cancer cells expressing *HORMAD1* more responsive to platinum‐based chemotherapy. Consistent with this, the use of PARP inhibitors on ovarian cancer stem cells induces foci of the meiosis‐specific RAD51 recombinase paralogue, DMC1 (Bellio *et al*., 2019). This could suggest that PARP inhibition induces an alternative homologous recombination pathway which is dependent on activation of meiotic homologous recombination mediators replacing the loss of somatic repair pathways and overriding the synthetic lethality caused by PARP inhibition.

In meiosis I, HORMAD1 is thought to ensure that DSBs are amenable to driving meiotic homologous recombination, a key feature of which is that it is biased down an inter‐homologue route, rather than an inter‐sister route. This is mediated by a number of meiosis‐specific factors including two factors, MND1‐HOP2 (Tsubouchi & Roeder, 2002; Chen *et al*., 2004). The first solid evidence that testis‐specific regulators of meiosis‐specific recombination could contribute to oncogenesis came from the finding that these two factors could drive the ALT (alternative lengthening of telomeres) mechanism in cancer cells (Arnoult & Karlseder, 2014; Cho *et al*., 2014b). To acquire the potential to become proliferative immortal, cancer cells must overcome the fact that telomeres become shorter each cell division due to the majority of somatic cells being deficient in active telomerase. Cancer cells not only can re‐activate telomerase, but also can initiate an ALT mechanism in the absence of this (De Vitis *et al*., 2018). ALT requires a recombination‐like mechanism to recognize the telomere end as a DSB and mediate the strand invasion of the end into a non‐homologous chromosome end; this strand invasion permits the initiation of a break‐induced DNA replication process to use the invaded non‐homologue telomeric DNA as a replicative template for the invading telomere to elongate (Dilley *et al*., 2016). A key enabler in this pathway is the need to bring non‐homologous ends into close proximity. It transpires that this enabler function is mediated by MND1‐HOP2 (Cho *et al*., 2014b). Not only was this the first clear demonstration of meiotic factors functioning in oncogenesis, but also the first example that reveals meiotic factors as potentially highly specific therapeutic targets for cancers.

### The meiotic cohesins in cancer

Chromosome regulation in both mitosis and meiosis is dependent upon the cohesin complex. In mitotically dividing cells, this complex serves to hold sister chromatids together following S phase through to the point at which the complex, and thus inter‐sister cohesion, is synchronously disrupted during the metaphase‐to‐anaphase transition, permitting the equational segregation of sister chromatids to opposing cellular poles in preparation for cytokinesis (Ishiguro, 2018). In addition to this role in sister chromatid cohesion, the complex also plays other fundamental roles, including functions in DSB repair and transcriptional regulation (Zhu & Wang, 2018). The cohesin complex forms a ring‐like structure which is thought to encircle both sister chromatids to mediate their cohesion and other functions (Rankin & Dawson, 2016). In meiosis, cohesin complexes can be distinguished from the mitotic cohesin complex as there are meiosis‐specific paralogues of some of the cohesin proteins that can replace their mitotic counterparts (Ishiguro, 2018). Given that meiotic cohesin complex mediates some very specialized meiosis‐specific functions, this interchangeability for cohesin subunits generates a potential weakness in the mitotic–meiotic regulatory interphase, as production of meiosis‐specific cohesin subunits in mitotic cells could readily impose oncogenic meiosis‐like functions on cells. One of the more prominent cohesin subunits that appears to be restricted to meiotic cells in many eukaryotes is REC8, one of the two paralogous counterparts to the RAD21 mitotic cohesin, RAD21L being the other (Ishiguro, 2018). REC8 not only is essential for inter‐homologue meiotic recombination, but also is needed for the establishment of sister centromere monopolarity in meiosis I, which is essential to ensure sister centromeres orientate to the same pole to drive the reductional segregation. To impose monopolarity onto sister centromeres in mitotically dividing cells could result in uniparent disomy (UPD). Recent seminal work has demonstrated that expression of a single meiotic cohesin subunit could indeed drive high frequencies of UPD in mitotically dividing cells (Folco *et al*., 2017). This work was carried out in the model organism *Schizosaccharomyces pombe* (fission yeast) and serves to irrefutably demonstrate the potential for imposition of meiotic cohesin function in mitotic cells by aberrant expression of only a single meiotic cohesin gene. In humans, this picture is less clear as expression of the human *REC8* gene does not appear to be restricted to the testes (Feichtinger *et al*., 2012; Uhlén *et al*., 2015), and indeed, there appears to be REC8 protein present in a range of non‐germ line somatic tissues (Uhlén *et al*., 2015). Clearly, this REC8 does not normally drive UPD in healthy human somatic cells, but these cells might be more readily primed to switch to a meiosis‐like function by as yet unknown cues. Interestingly, REC8 does not appear to be incorporated into mitotic cohesin complex in HEK293 cells unless another meiosis‐specific cohesion subunit, STAG3, is activated (Wolf *et al*., 2018); so, potentially in human somatic cells REC8 meiotic function becomes activated by the aberrant expression of other meiotic‐specific genes. Importantly, a reported feature of some female GC cancers is homozygosity, postulated to be caused by a failure of meiosis II or endoreduplication (Kato *et al*., 2018). However, another possibility to be considered is that this is driven by UPD caused by sister centromeres taking on a meiosis I‐like monopolar configuration, possibly mediated by a meiosis‐specific function of REC8 being activated.

Despite REC8 appearing to be present in normal somatic tissues, oncogenic activity has been ascribed to it. This includes inference of a pseudomeiotic role for REC8 in endoploid cells induced after ionizing irradiation (Erenpreisa *et al*., 2009) and the finding that REC8 might be a tumour suppressor required to inhibit the epithelial‐to‐mesenchymal transition in gastric cancers, possibly via a transcriptional regulation function (Yu *et al*., 2017; Zhao *et al*., 2018).

Other meiosis‐specific cohesin subunits, which have gene expression tightly restricted to the testis in healthy humans, are STAG3, SMC1β and RAD21L, the latter being another RAD21/REC8 paralogue (Ishiguro, 2018). The corresponding genes encoding these cohesins have been found to be activated in some cancers, and overexpression of *RAD21L* in murine primary fibroblasts induces increased adjacency of homologous chromosomes (Feichtinger *et al*., 2012; Rong *et al*., 2017). This could help to facilitate oncogenic drivers such as ALT (see above) and induction of loss of heterozygosity via homologous recombination‐mediated repair of DSBs via an inter‐homologue route, suggesting that *RAD21L* expression could be oncogenic. STAG3 levels have also been linked to resistance of inhibition of the BRAF oncogene activity in melanoma (Shen *et al*., 2016). The mechanism of this is unknown, but it is reduced levels of *STAG3* that are associated with resistance, suggesting that *STAG3* expression is a sensitizer, which suggests elevating expression of a meiotic cohesin gene is a distinct therapeutic avenue in combination with BRAF inhibition (Shen *et al*., 2016).

## Meiotic Factors in Testicular Germ Cell Tumours: Meiosis or Pseudomeiotic Activation?

During embryonic development, PGCs migrate to the genital ridge and enter the developing gonad, where they are directed towards alternate pathways to differentiate into either oogonia or spermatogonia (Jørgensen & Rajpert‐De Meyts, 2014). This either leads to entering meiosis in the foetal ovaries or leads to the inhibition of meiosis initiation in the testes (Spiller & Bowles, 2015). The switch between mitosis and meiosis is a fundamental and tightly controlled feature that occurs during germ cell development, and even though it has not caught much attention of GC tumour research, dysregulation of meiosis is believed to play a role in GC tumour development (Jørgensen *et al*., 2013; Lanza & Heaney, 2017).

Testicular germ cell tumours (TGCTs) are currently classified into tumours that are derived from germ cell neoplasia *in situ* (GCNIS, type II) and into those that are not (non‐GCNIS, types I and III) (Williamson *et al*., 2017). The non‐GCNIS tumours are subdivided into type I (teratoma and yolk sac tumour) and type III (spermatocytic tumours) malignancies. GCNIS‐derived, type II tumours can be divided into two main histologic types, seminomatous and non‐seminomatous TGCTs, with non‐seminomatous types further grouped into embryonal carcinoma, yolk sac tumour, teratoma and choriocarcinoma (Looijenga & Oosterhuis, 2002; Williamson *et al*., 2017). TGCTs are not believed to derive from a mature germ cell but rather to arise from PGCs (Oosterhuis & Looijenga, 2005) and hence have their origin during embryogenesis (Pierpont *et al*., 2017). A current tumour evolution model suggests that non‐GCNIS‐derived, type I GC tumours arise in the early stages of germ cell development during PGC migration and proliferation, whereas GCNIS‐derived tumours are preceded by GCNIS cells that originate from gonadal PGCs (Cheng *et al*., 2018; Pierce *et al*., 2018). During germ line development, PGCs undergo extensive epigenetic reprogramming, including modification of histone marks and erasure of imprinting, which is thought to prepare the cells for differentiation (Sato *et al*., 2003; Seki *et al*., 2005; Kristensen *et al*., 2013; Hill *et al*., 2018). Consistent with the suggestion that the cell of origin for type I neoplasms is of earlier stage, the type I GC tumours have been found to be partially imprinted and type II seminomas resemble gonadal PGCs in their erased DNA methylation pattern (Ross *et al*., 1999; Schneider *et al*., 2001; Shen *et al*., 2018). Type II non‐seminomatous TGCTs, however, appear to have regained some methylation patterns (Shen *et al*., 2018). Also, an investigation of genetic and epigenetic alterations in paediatric GC tumours concluded that most studied tumours derived from pre‐meiotic PGCs and only in a few cases tumours appear to have arisen from errors during meiosis (Ichikawa *et al*., 2013), although the UPD observed in some of these tumours could also be explained by aberrant development of monopolar centromeres by activation of REC8, for example (see above), and not by a meiotic error per se. Only the rare type III malignancies originate from more mature post‐natal cells (although probably from cells transitioning from spermatogonia to spermatocytes, which are still pre‐meiotic cells) (Rajpert‐De Meyts *et al*., 2003; Giannoulatou *et al*., 2017).

**Figure 4:**
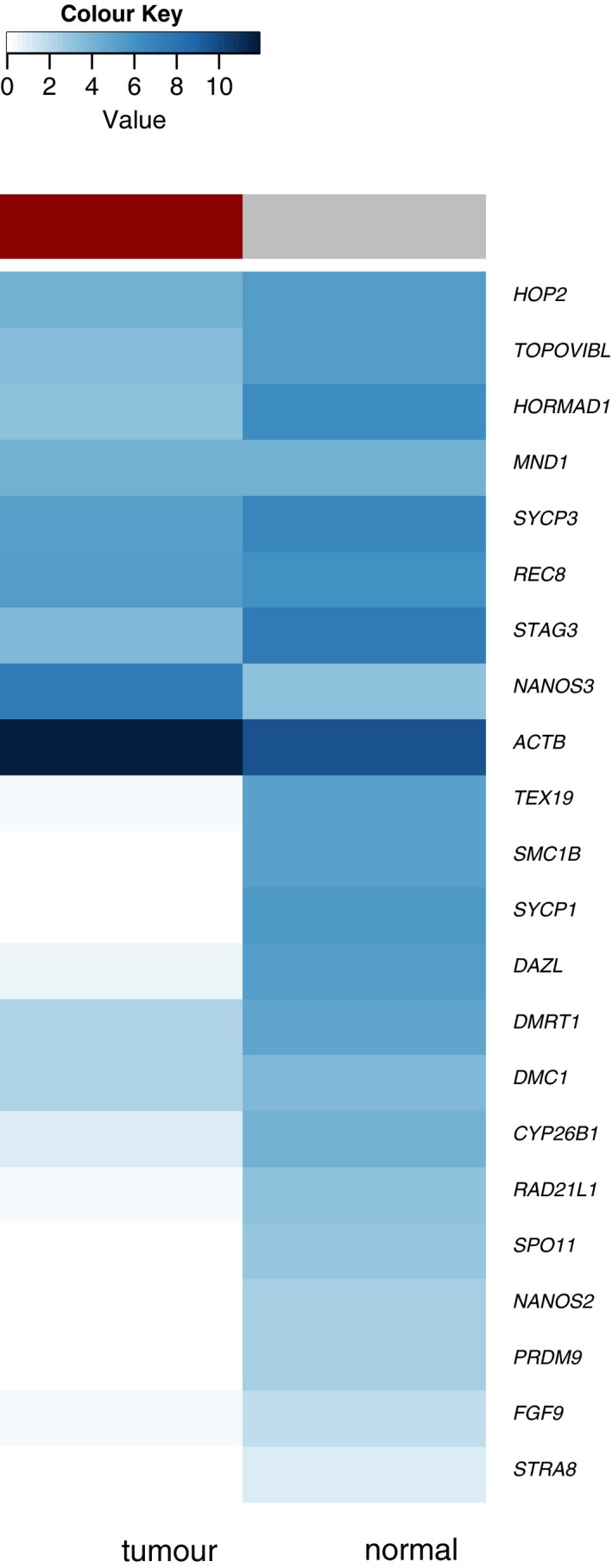
Median expression of selected meiotic entry and meiotic chromosome regulatory genes in TCGA testicular germ cell cancers (TCGA‐TGCT) and GTEx normal testis (excluding capsule). Expression presented as log2 (transcripts per million + 1) values, which were extracted from the GEPIA database (Tang *et al*., 2017) and plotted as heatmap in R (the range from white to dark blue represents increasing expression values). *ACTB* was included to show expression relative to a housekeeping gene. The TCGA‐TGCT data set is derived from 137 tumours [72 seminoma, 18 embryonal carcinoma (EC), nine EC dominant, three mature teratoma, 10 mature teratoma dominant, three immature teratoma dominant, five yolk sac tumours, eight yolk sac tumours dominant and nine mixed tumours with no dominant component (dominant > 60% presence of a given histology)] (Shen *et al*., 2018). In TGCTs, full expression of meiotic programme is clearly absent and aberrant activation of meiotic genes from various stages appears to have occurred. In normal testis, however, expression of the full meiotic programme can be observed, as this includes the expression profiles of the full spectrum of pre‐meiotic, meiotic to post‐meiotic cells.

Whilst the cells of origin for the various tumour types have somewhat been pinpointed, it is not clear how these tumour cells maintain a proliferative programme. In a normal developmental setting, PGCs at extragonadal sites as well as at the developing ovaries enter meiosis (McLaren & Southee, 1997), whereas entry into mitotic G1/G0 arrest occurs in the developing testes; yet, germ cells of TGCT‐susceptible mice were associated with delayed entry into mitotic arrest and retention of pluripotency (Heaney *et al*., 2012). Furthermore, it is thought that GCNIS and tumour cells are not able to progress through meiotic prophase I, as impaired meiotic competence has been observed in cells with PGC characteristics derived from mouse embryonic stem cells (Tedesco *et al*., 2011; Jørgensen *et al*., 2013). This raises the question whether these cells are mitotic and proliferating or attempt to enter meiosis but fail to proceed and revert to mitotic proliferation through a defective mitotic–meiotic switch, rendering them sexually confused. Indeed, a study by Jørgensen co‐workers reported meiosis‐inducing and meiosis‐inhibiting factors to be expressed simultaneously in GCNIS (Jørgensen *et al*., 2013), supporting their suggestion of sexually confused cells (Rajpert‐De Meyts, 2006). Further support is provided by the fact that patients with disorders of sex differentiation exhibit a higher risk of developing GC tumours (Skakkebaek *et al*., 2016). Yet another possibility is that most GC tumours derive from pre‐meiotic PGCs, in which meiotic factors are prematurely and unscheduled activated through defects in gene regulatory mechanisms as observed in other cancers, rather than through a defect in the switching between the mitotic and meiotic modes. As discussed in the previous section, many tumours exhibit aberrant expression of meiotic genes, in particular meiotic chromosome regulators (and germ line genes in general), and oncogenic links have been shown (McFarlane & Wakeman, 2017). Either way, these cells do not or cannot initiate a full meiotic programme, which is supported by the fact that varying and contradicting expression patterns of meiotic genes in GC tumours, cell lines and/or pre‐malignant GCNIS have been reported (Adamah *et al*., 2006; Jørgensen *et al*., 2013) (Fig. 4).

In relation to GC tumours, mostly meiotic entry regulator genes have been studied, as the main focus was laid on meiotic entry and the dysregulation of the mitotic–meiotic switch. Using TCGA Research Network (http://cancergenome.nih.gov/) and GTEx (Carithers *et al*., 2015; Keen & Moore, 2015) data extracted through the GEPIA database (Tang *et al*., 2017), we assessed the expression of a number of selected meiotic entry and meiotic chromosome regulatory genes – hence genes expressed at various stages of meiosis (Fig. 4). Interestingly, in TGCTs, full expression of meiotic programme is clearly absent. Indeed, aberrant activation of meiotic genes from various stages appears to have occurred, including the conflicting expression of meiosis‐inducing and meiosis‐inhibiting factors, as reported before (Jørgensen *et al*., 2013), but also the expression of meiotic chromosome regulators as reported in other cancers (McFarlane & Wakeman, 2017). In normal testis, however, expression of the full meiotic programme can be observed, as this includes the expression profiles of the full spectrum of pre‐meiotic, meiotic to post‐meiotic cells. Recently, single‐cell RNA‐sequencing experiments dissected the progression through meiosis (Chen *et al*., 2018; Guo *et al*., 2018). According to Guo *et al*. (2018), the selected meiotic genes aberrantly activated in TGCTs (Fig. 4) should not be expressed at the same time.

Often markers for meiotic entry and meiosis (e.g. Spiller *et al*., 2012) are used in studies but should be interpreted critically, particularly in a pathological setting, as they could represent an unscheduled aberrant activation. For example, a study by Heaney co‐workers showed that only in rare cases, aberrant expression of *STRA8* induced entry into meiosis (Heaney *et al*., 2012) and is, therefore, not a reliable marker for meiotic entry. Neither is *SYCP3* expression suitable as meiotic entry marker without interrogating the functional activity of the SYCP3 protein, such as chromosomal association and further formation of the SC, or showing expression of genes in the associated meiotic gene cluster (Heaney *et al*., 2012; Guo *et al*., 2018). Aberrant, unscheduled *SCYP3* expression outside the meiotic context has been shown in other cancers (Chung *et al*., 2013; Cho *et al*., 2014a; Kitano *et al*., 2017), where it has the potential to disrupt the activity of BRCA2, potentially leading to the modulation of strand invasion capabilities for recombinases (RAD51 and DMC1), which in turn drive homologous recombination (Hosoya *et al*., 2011; Kobayashi *et al*., 2017) (see above). Moreover, GC tumour research has been mainly conducted in the mouse model and results should be cautiously translated into a human setting. As mentioned previously, the expression of the human *REC8* gene, for example, does not appear to be restricted to the testes (Feichtinger *et al*., 2012; Uhlén *et al*., 2015), whereas it is indeed meiosis‐specific in mice.

## Concluding Remarks

Although solid tumours are characterized by heterogeneity, cancer cells exhibit certain stereotypical functional capabilities, such as growth and proliferative advantages, altered stress response, invasion and metastasis, stimulated angiogenesis and immune evasion (Fouad & Aanei, 2017). Nonetheless, how cancer cells acquire such malignant characteristics remains very poorly understood. One view is the embryologic theory of cancer, in which cancer cells acquire embryo‐like characteristics that would explain the malignant features of cancer cells (Erenpreisa *et al*., 2015). To some degree, this altered, or confused, developmental model has been applied to interpret the expression of meiotic genes in GC tumours. However, a range of cancers express distinct subsets of genes from various otherwise restricted programmes such as meiotic/germ line and placental/embryogenesis‐related to a varying degree (Jungbluth *et al*., 2007; Macaulay *et al*., 2017; McFarlane & Wakeman, 2017; Bruggeman *et al*., 2018; Costanzo *et al*., 2018). Hence, it could be speculated that cancer cells acquire their malignant characteristics by aberrant activation of a single gene or cluster of genes from one or more of these programmes, and this occurs as it confers a selective advantage on the cancer cell. Given that GC tumours arise from cells intimately associated, both temporally and spatially, with the meiotic developmental programme, current data do not permit a clear delineation of why meiotic factors become activated in some of these tumours and what role they play. The core question of whether activation of these factors is linked to a developmental confusion or positive selective activation (or both in distinct GC tumour subtypes) appears to remain unaddressed. What is clear is that an open view of the causes and consequences of meiotic gene activation in GC tumours should be applied, as this may yet reveal new and important aspects of GC tumour biology and new therapeutic avenues.

## Meeting Comments

### Niels Skakkebaek (Copenhagen, Denmark)

Are the meiosis‐specific genes the same in males and females?

### Ramsay McFarlane

That is not known for certain but we assume that the core proteins driving meiosis are the same in both sexes. However, there are clearly differences between the sexes. One example is the meiosis‐specific protein SPO11, which mediates the formation of meiosis‐specific DNA double‐strand break formation. In males there is a specific variant of SPO11 which serves to mediate XY inter chromosome associations in the pseudo autosomal region. This function does not occur in females.

### Ewa Rajpert‐De Meyts (Copenhagen, Denmark)

Your studies are possibly related to the mechanisms of malignant transformation of germ cells in males and females. The fetal testis has an intricate mechanism with several backups to prevent meiosis in germ cells, and this is regulated by their somatic niche. Such regulation is absent in the fetal ovary where meiosis occurs very early in life. The first stage of malignant transformation of testicular germ cells is the formation of GCNIS cells, and these cells often display premature aberrant partial activation of meiosis. This would be expected to hamper growth of such cells, but somehow it appears to help the abnormal cells to survive and proliferate.

### Ramsay McFarlane

Our initial thoughts were that premature activation of meiosis must have negative consequences for the cell, and one might assume that these would be anti‐proliferative. I think, however, that evidence from a range of cancers is showing that this view is incorrect, as a number of meiotic genes can contribute oncogenic functions to drive the proliferative state. How this relates to germ line cancers remains an open question, in my view. Is meiotic gene activation in germ line tumours a real attempt to start a full meiotic program, as one might assume, given the nature of the cells? Or, is it simply due to activation of some meiotic genes conferring a selective proliferative advantage (or some combination of both)? I think this is an interesting and open question and we should keep an open mind and look to generate data to support or refute all options.

### Leendert Looijenga (Rotterdam, the Netherlands)

In the NTERA‐2 cell line with TEX19 knock down, did you exclude that you were not only stopping proliferation, but perhaps you were simply seeing cell differentiation?

### Ramsay McFarlane

The NTERA‐2 cell line is capable of undergoing differentiation. Treatment with retinoic acid (RA) causes differentiation along a neuronal lineage whereas HMBA treatment results in differentiation down ill‐defined lineages. We assessed cell morphology changes and *OCT4* expression following TEX19 knockdown to address whether reduction in TEX19 levels triggered differentiation; we could find no overt evidence that differentiation was occurring. We used both HMBA and RA treated NTERA‐2 cells as controls. We also assessed what happens to *TEX19* expression following differentiation induced by either RA or HMBA and found that whilst *OCT4* RNA levels were reduced quite quickly, *TEX19* transcript levels stayed relatively high until complete differentiation, suggesting there is not an obvious correlation to OCT4.

### Tim Oliver (London, UK)

You mentioned the *BRCA2* gene, but have you looked at other DNA repair genes such as *TP53*, which possibly explains the sensitivity of seminoma to treatment?

### Ramsay McFarlane

We have not studied BRCA2 directly in our experiments although others have demonstrated that the meiosis‐specific SYCP3 protein can disrupt BRCA2 function. Whilst I'm not aware that this has been studied extensively in germ line tumours, S*YCP3* does become expressed in germ line tumours, so disruption of BRCA2 activity in germ line tumours by meiotic factors is an interesting possibility.

No we have not explored a relationship between other meiotic factors and TP53 in germ line tumours.

### Finn Edler von Eyben (Odense, Denmark)

In meiosis, there is a reduction of chromosomes from 2N to 1N, whereas, in testicular tumours there is duplication of chromosomes from 2N to 4N. Can you speculate what is driving this change?

### Ramsay McFarlane

This is possible premeiotic replication without subsequent segregation. Alternatively, there are other pathways that could cause ploidy changes due to meiotic gene activation, not least of which is the aberrant formation of mono polar centromeric associations between sister centromeres by the activation of REC8 cohesin function – this would explain the centromeric homozygocity observed in some germ line tumours.
